# Mild repetitive head impacts alter perivascular flow in the midbrain dopaminergic system in awake rats

**DOI:** 10.1093/braincomms/fcab265

**Published:** 2021-11-03

**Authors:** Xuezhu Cai, Ian C Harding, Aymen H Sadaka, Bradley Colarusso, Praveen Kulkarni, Eno Ebong, Ju Qiao, Nick R O'Hare, Craig F Ferris

**Affiliations:** 1 Department of Psychology, Center for Translational NeuroImaging, Northeastern University, Boston, MA 02115, USA; 2 Department of Bioengineering, Northeastern University, Boston, MA 02115, USA; 3 Department of Chemical Engineering, Northeastern University, Boston, MA 02115, USA; 4 Department of Neuroscience, Albert Einstein College of Medicine, New York, NY 10461, USA; 5 Department of Psychology, Northeastern University, Boston, MA 02115, USA; 6 Department of Pharmaceutical Sciences, Northeastern University, Boston, MA 02115, USA

**Keywords:** substantia nigra, ventral tegmental area, microglia, end-feet, neuroinflammation

## Abstract

Head injury is a known risk factor for Parkinson’s disease. Disruption in the perivascular clearance of metabolic waste and unwanted proteins is thought to be a contributing factor to disease progression. We hypothesized that repetitive mild head impacts, without evidence of structural brain damage, would increase microgliosis and AQP4 expression and depolarization and alter perivascular flow in the midbrain dopaminergic system. Adult male rats were subjected to sham, or two mild head impacts separated by 48 h. Three weeks later, fully awake rats were imaged using dynamic, contrast-enhanced MRI to follow the distribution of intraventricular gadobenate dimeglumine contrast agent. Images were registered to and analysed using a 3D MRI rat atlas providing site-specific data on 171 different brain areas. Following imaging, rats were tested for cognitive function using the Barnes maze assay. Histological analyses of tyrosine hydroxylase, microglia activation and AQP4 expression and polarization were performed on a parallel cohort of head impacted rats at 20 days post insult to coordinate with the time of imaging. There was no change in the global flux of contrast agent between sham and head impacted rats. The midbrain dopaminergic system showed a significant decrease in the influx of contrast agent as compared to sham controls together with a significant increase in microgliosis, AQP4 expression and depolarization. There were no deficits in cognitive function. The histology showed a significant level of neuroinflammation in the midbrain dopaminergic system 3 weeks post mild repetitive head impact but no loss in tyrosine hydroxylase. MRI revealed no structural brain damage emphasizing the potential serious consequences of mild head impacts on sustained brain neuroinflammation in this area critical to the pathophysiology of Parkinson’s.

## Teaser

Cai et al. report that mild head impact without evidence of brain damage can alter perivascular clearance and increase neuroinflammation around the dopaminergic neurons in the midbrain substantia nigra weeks after injury. These data support the notion that early head injury may be a risk factor for Parkinson’s disease.

## Graphical Abstract

**Graphical Abstract:**
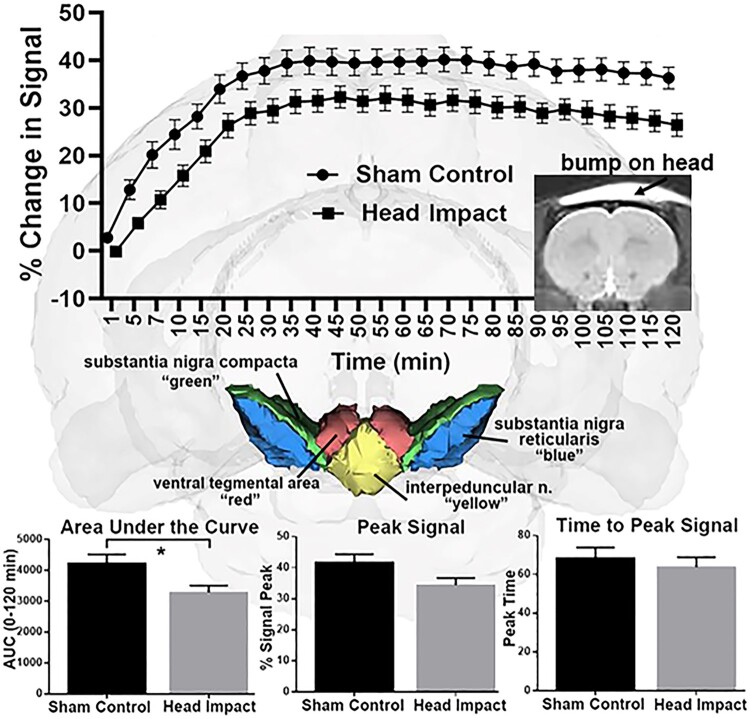

## Introduction

Parkinson’s disease is the second most common neurodegenerative disorder after Alzheimer’s and affects nearly 1 in 1000 people globally.^1^ It is a disease of ageing combined with genetic and environmental risks.^2^ Traumatic brain injury (TBI) is one of the environmental risk factors contributing to the pathogenesis of Parkinson’s disease.^3–7^ There are numerous preclinical studies reporting damage to the midbrain dopaminergic system (MDS) after moderate to severe TBI.^8–14^ The damage is characterized by loss of dopamine (DA) neurons in the substantia nigra (SN), increased accumulation of α-synuclein aggregates and putative Lewy bodies, and neuroinflammation marked by activated microglia. Repetitive mild head injury without structural brain damage reduces neural coupling to the SN as measured with resting-state functional connectivity several weeks post head impact.^15^

There is a growing literature that perivascular flow and AQP4 may play a critical role in clearing beta amyloid and tau in Alzheimer’s disease and TBI.^16–18^ The glymphatic system has also been hypothesized to have a purported role in the clearance of metabolic toxins and α-synuclein in the midbrain that maybe contributing to the aetiology and pathophysiology of Parkinson’s disease.^19^^,^^20^ Studies on awake rats show the SN has a significant diurnal variation in perivascular clearance of contrast agent across the light dark cycle with the highest efflux occurring during the light cycle when rats are at rest or sleeping.^19^ Zou et al.^21^ working with A53T mice overexpressing mutated human α-synuclein showed that blocking meningeal lymphatic drainage increases intra and extracellular aggregation of α-synuclein protein in the SN together with the insoluble oligomeric form of the protein. They also reported that blocking lymphatic drainage enhances AQP4 depolarization and neuroinflammation in the SN of these transgenic mice.

While brain injury is a well-documented risk factor for Parkinson’s disease affecting the chemistry and function of the MDS, and while the clearance of metabolic toxins and α-synuclein from this area is a likely contributing factor to the disease, there have been no studies combining mild head injury with perivascular clearance at the level of the MDS. We have hypothesized that mild repetitive head impact would disrupt perivascular clearance in the MDS, and that this area would show altered AQP4 expression and polarization and increased neuroinflammation. Indeed, 3 weeks following only two mild head impacts without evidence of structural brain damage or contusion, there was a reduction in accumulation of contrast agent in the MDS together with an increase in microgliosis and altered AQP4 levels. These observations have been interpreted as a neuroadaptive response to sustained neuroinflammation and vasogenic oedema.

## Methods

### Animals

Thirty-eight Sprague Dawley male rats (250–300 g) were purchased from Charles River Laboratories (Wilmington, MA, USA), housed on a 12:12 light–dark cycle (lights on at 7:00 a.m.) maintained at ambient temperature (22–24°C) and provided with food and water ad libitum. Twenty-six rats were randomly assigned to the perivascular imaging study followed by behaviour assessment, while 12 rats were assigned to the immunohistochemical analysis designed to coincide with the timing of the imaging. All animals were cared for in accordance with the NIH Guide for the Care and Use of Laboratory Animals. Methods and procedures used in this study were pre-approved by the Northeastern University Institutional Animal Care and Use Committee The protocols used in this study complied with the regulations of the Institution and adhered to the ARRIVE guidelines for reporting *in vivo* experiments in animal research.^22^

### Acclimation for awake imaging

**Figure 1:**
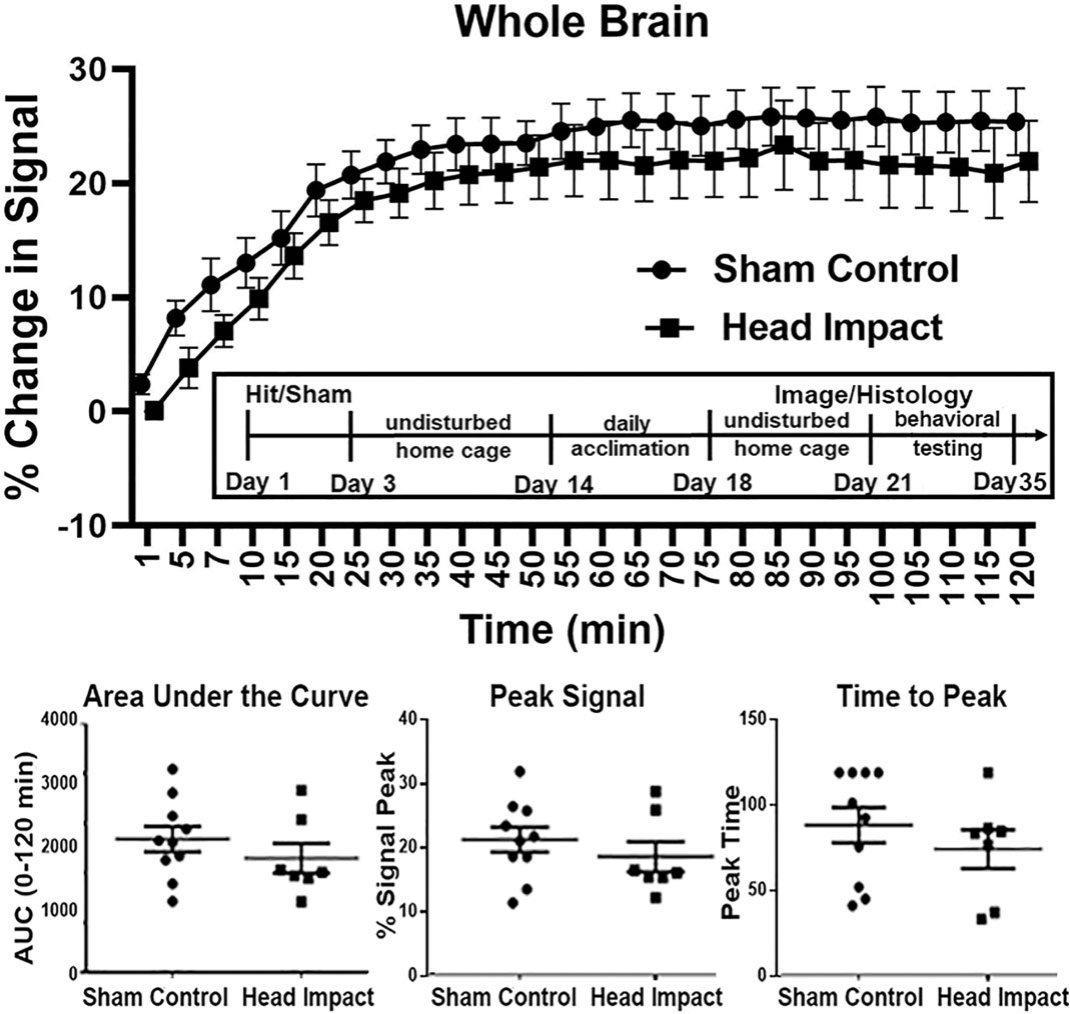
**Time-to-signal curve in whole brain.** *Top panel*: Awake DCE-MRI shows the contrast agent kinetics following ICV injection over a 2-h period in the whole brain. Two-way ANOVA identified no significant difference between the two groups [*P* = 0.3332, *F*(1,15) = 0.9997]. Group sizes: Sham Control (*n* = 10); Head Impact (*n* = 7). The box insert shows a timeline of experimental procedures. *Bottom panel*: Dot plots of extracted CA kinetic parameters, including AUC, peak signal, and time to peak signal for the sham control and head impact groups. The parameters extracted from the fitting curves were compared using two-sample *t*-test. No significance was observed for overall perivascular flow in the two experimental groups.

The use of anaesthesia to study the glymphatic system has been a subject of some debate following the publication by Gakuba et al. showing data that anaesthesia impairs flow.^23^ However, a detailed study testing several anaesthetic formulae combined with EEG and cardiorespiratory measures reported a mixed effect on movement with some anaesthetics like ketamine/xylazine enhancing movement while high dose isoflurane reduced movement.^24^ To avoid the confound of anaesthesia, movement along the perivascular space was evaluated in fully awake rats. Two weeks following sham (*n* = 13), or mild head impact (*n* = 13) rats were prepared for awake imaging, by exposing them to daily acclimation sessions over five consecutive days. Rats were lightly anaesthetized with 1–2% isoflurane (box followed by nose cone) and placed into the restraining system used during imaging. When fully conscious, the rats were placed into a dark mock scanner tube with a sound recording of a standard MRI pulse sequence playing in the background. This acclimation procedure has been shown to significantly reduce plasma corticosterone, respiration, heart rate and motor movements when compared to the first day of acclimation. The reduction in autonomic and somatic response measures of arousal and stress improve the signal resolution and MR image quality.^25^ Interestingly, five of the rats from the head impact group failed to acclimate, i.e. continued to struggle and escape from the passive head holding device (see below) while only one rat from the sham group failed to acclimate. We normally see a 10% attrition in animal numbers during acclimation for awake imaging.^26^ In addition, one rat from the head impact group died during surgery for placement of the injection cannula into the lateral cerebroventricle, while two rats from the sham group were eliminated from the study due to misplacement of the injection cannula. The final group numbers were sham (*n* = 10) and head impact (*n* = 7). A timeline of experimental procedures is included in Fig. 1. On Day 1 and Day 3, rats were hit or sham treated and left undisturbed in their home cage until Day 14. Between Day 14 and Day 18, rats were acclimated to the awake imaging procedure. On Day 21, 3 weeks following the first hit rats were imaged. A parallel group of 12 rats (see Immunohistochemistry below) were treated the same way except on Day 21 they were not imaged but euthanized for histopathology. Rats that were imaged were followed for another 14 days during which they were tested for behaviour.

### Momentum exchange model

Head impacts were generated using a closed head, momentum exchange model previously described.^27^^,^^28^ The kinetic energy at impact was 1.37 joules.^29^ We have used this model to publish on the neuroradiological effects of a single mild head impact,^30^ multiple mild impacts^31^ and moderate brain injury in rats.^32^ The impact piston was directed to the top of the skull, midline, in the approximate area of Bregma (see Supplementary Fig. 1A). There was no evidence of structural brain damage or contusion in any of the impacted rats (see Supplementary Fig. 1B). All sham controls and impacted rats were anaesthetized with 2% isoflurane. Rats were awake and ambulatory within 5–7 min after anaesthesia and head impact. Forty-eight hours after the first head impact rats were again anaesthetized and subjected to a second head impact or sham procedure. Three weeks later, fully awake sham and hit rats were imaged using dynamic, contrast-enhanced MRI to follow the distribution of intraventricular gadobenate dimeglumine contrast agent as described below.

### Surgical procedure

Just prior to imaging, rats were anaesthetized with 2–3% isoflurane and received a subcutaneous injection of the analgesic Metacam (Meloxicam, 5 mg/mL solution) in a dose of 1 mg/kg. The scalp was incised, and a burr hole made in the skull for implantation of sterile PE10 tubing (Braintree Scientific) aimed at the right lateral cerebroventricle using the stereotaxic coordinates: 1.0 mm posterior to the bregma, 2.0 mm lateral to the midline, and 4.0 mm in depth from dura as previously described.^19^ Tubing prefilled with gadobenate dimeglumine (MultiHance^®^) 1.06 kD, contrast agent diluted at 1:20, was fixed in place with cyanoacrylic cement allowing for infusion outside the bore of the magnet. This injection method has been used in previous studies to deliver drugs directly to the brain during awake imaging.^33^^,^^34^

### Imaging acquisition

Rats were imaged within the first 4 h of the onset of the light–dark cycle between 7:00 and 11:00 h. MR imaging was performed on a Bruker BioSpec 7 T/20 cm USR MRI spectrometer controlled by Paravision 6.0 software as previously described.^19^ Radio frequency signals were sent and received with a custom quadrature volume coil built into the animal restrainer (Ekam Imaging, Boston, MA, USA). Immediately after surgery, rats were quickly placed into the head coil and restraining system, a procedure that takes less than a minute (www.youtube.com/watch?v=JQX1wgOV3K4). The design of the restraining system includes a padded head support obviating the need for ear bars helping to reduce animal discomfort while minimizing motion artefact.

### Imaging analysis

Image pre-processing and analysis were performed with a combination of 3D slicer, AFNI, FSL, MIVA and MATLAB software. Reconstructed T_1_-weighted image data were rescaled to the original intensity measurements (divided by the receiver gain and multiplied by the scaling factor called SLOPE) for each time point for each subject. The signal intensities extracted from manually drawn ROIs in the CA phantom at different time points were used for validation. Head motion correction was conducted for the first sequential acquisition session with the middle volume as the reference image using the AFNI program. MR images were skull-stripped semi-automatically by applying a manually drawn brain mask of the reference image to co-registered 4D concatenated anatomical data. Spatial smoothing with FWHM = 0.2 mm was performed on all images for each rat. A 3D MRI Rat Brain Atlas © (Ekam Solutions LLC, Boston, MA) containing 171 brain regions was used for brain segmentation for each subject by manually registering the atlas to the subject space using a rigid registration method. Subsequent analysis of perivascular clearance for each ROIs was conducted as previously described.^19^

### Statistical significance

Statistical analyses were performed using Prism 8 (Graphpad, La Jolla, CA, USA). Raw data points are presented as dot plots and two-way ANOVAs were used for comparison with time points being a within-subject factor and head injury being a between-subject factor, followed by a Sidak multiple correction. The parameters extracted from the fitting curves were compared using two-sample *t*-test. *P* < 0.05 was considered statistically significant. The peak signal and AUC were acquired from the fitting curves based on the original data points and compared using a *t*-test. Missing data points or data of insufficient quality were replaced by values from the fitting curve with random noise.

### Behaviour

The rats used in the imaging study were then studied for changes in cognitive behaviour using the Barnes Maze, a protocol that takes several days to complete for each rat extending the time between the end of imaging and completion of testing by 2–3 weeks. A description of the Barnes maze and subsequent data analysis has been reported in previous studies from our laboratory.^32^^,^^35^^,^^36^ All trials were video recorded and analysed using manual methods by experimenters blind to treatment condition and verified with automated scoring using ANY-maze^®^ software (Stoelting, Wood Dale, IL). All behaviour testing was conducted between 0800 and 1600 in a humidity and temperature-controlled facility.

#### Behaviour statistics

All analyses were completed by researcher’s blind to experimental manipulations. One-way or Mixed design analysis of variance (ANOVA), followed by Tukey’s *posthoc* comparison of 95% CI (multiplicity adjusted *P*-values reported), were performed where appropriate, unless otherwise noted. All alpha levels were set to 0.05. GraphPad Prism version 6.0 (GraphPad Software, La Jolla, CA) was used for all statistical analyses, unless otherwise noted. Heatmaps where constructed using custom *Mathematica* (Wolfram Research, Champaign, IL).

### Immunohistochemistry

At 20 days post insult, a separate group of 12 rats (sham *n* = 6) and (head impact *n* = 6) were anaesthetized with 3% isoflurane, and transcardially perfused with PBS followed by 4% paraformaldehyde (PFA) solution. The timing of the tissue collection was designed to coincide with the timing of the imaging. After brains were extracted from the subjects, brain samples were further fixed in 4% PFA for 24 h and subsequently preserved in 30% sucrose in PBS at 4°C. Brain tissues were then sectioned using a cryomicrotome with a thickness of 40 microns. To prepare brain tissue for immunohistochemistry, prefrontal cortex and midbrain sections of the brain tissue were collected in six wells repeatedly in sequential order with 240 µm intervals between each section in each well. Sections were placed in a glycine solution and stored at −20°C.

Free-floating immunohistochemistry was conducted on animals post MR imaging to assess neuroinflammation, by staining microglia-specific ionized calcium binding adaptor molecule 1 (Iba1), cerebrospinal fluid and interstitial fluid (CSF–ISF) exchange, by staining astrocytic AQP4 water channels, impairment of midbrain dopaminergic function, by staining for the tyrosine hydroxylase (TH) enzyme responsible for dopamine production. For Iba1 and Aqp4 staining, brain sections were blocked with 5% goat serum in 0.1% PBST for 1 h and subsequently incubated overnight at 4°C with primary antibody: anti-Iba1 (1:1000 in 5% goat serum and 0.2% PBST, WAKO, cat #019-19741). The sections were washed three times in 0.1% PBST for 5 min each. Sections were then incubated with secondary antibody (1:500, goat anti-rabbit, Alexa Fluor 488) in PBS for 1 h at room temperature and mounted with Vectashield Mounting Medium with DAPI (Vector laboratories). Double immunofluorescence labelling of AQP4, and blood vessels was carried out using Atto-594 conjugated anti-AQP4 antibody (1:500) and DyLight 488 conjugated Lycopersicon Esculentum (Tomato) Lectin (1:100, Vector Laboratories, cat # DL-1174), respectively. Sections were incubated in 0.2% PBST overnight at 4°C and mounted with Vectashield.

For TH staining, sections were first blocked/permeabilized in 0.4% Triton X-100, 5% goat serum in PBS for 1 h at room temperature and subsequently incubated with an anti-TH primary antibody (1:250 dilution; ThermoFisher; cat# PA5-85167) diluted in 0.1% Triton X-100, 5% goat serum in PBS overnight at 4°C. Following primary antibody incubation, the sections were washed with PBS and incubated in the species appropriate secondary antibody (1:1000) for 1 h at room temperature, washed, and then mounted with Vectashield. To quantify dopaminergic function, the expression of TH in the substantia nigra was measured by calculating the mean fluorescence intensity of TH signal within the substantia. At least three individual sections were analysed per subject.

The interpeduncular n. was included in the midbrain dopaminergic system together with the ventral tegmental area and substantia nigra compacta and reticularis. While the body and ventral aspect of the interpeduncular n. has little tyrosine hydroxylase (TH) staining the dorsal adjacent area of the interfascicularis n. and lateral dorsal paranigra n. are rich in TH positive cell bodies and fibers^37^ and are included in the boundaries of the rat 3D MRI atlas.

Images used for quantification were collected on a Zeiss LSM 880 confocal microscope using ZEN Black software. *Z*-stacks were collected in 3 micron increments over a span of approximately 30 microns for a total of ∼10 sections located between −2.8 and −3.8 mm from the bregma, depending on the subject and sections collected. All analysis was performed by researchers blind to experimental manipulations. The data were statistically evaluated through Prism software (Graphpad, La Jolla, CA, USA). All data sets were initially confirmed for normal distributions (Anderson-Darling normality test) and equal variances (Bartlett’s and Levene’s test for homogeneity). Comparisons between values for each condition were made with *t*-tests.

### Data availability

All data can be accessed through a link to Mendeley. DOI: 10.17632/dsz7j9sjgw.1.

## Results

Shown in Fig. 1 are time series for changes in signal contrast for the whole brain following ICV injection of trace in rats with a history of two head impacts (black circles) or sham controls (black boxes) 20 days post insult. There was no significant difference between experimental conditions by 2-Way ANOVA [*F*(1,15) = 0.999, *P* = 0.333]. The bar graphs below showing measures of AUC (*P* = 0.340), peak signal (*P* = 0.395) and time to peak (*P* = 0.382) were not significantly different between conditions by a two-tailed *t*-test. Why is Fig 1 presented in the Methods and not here?

**Figure 2:**
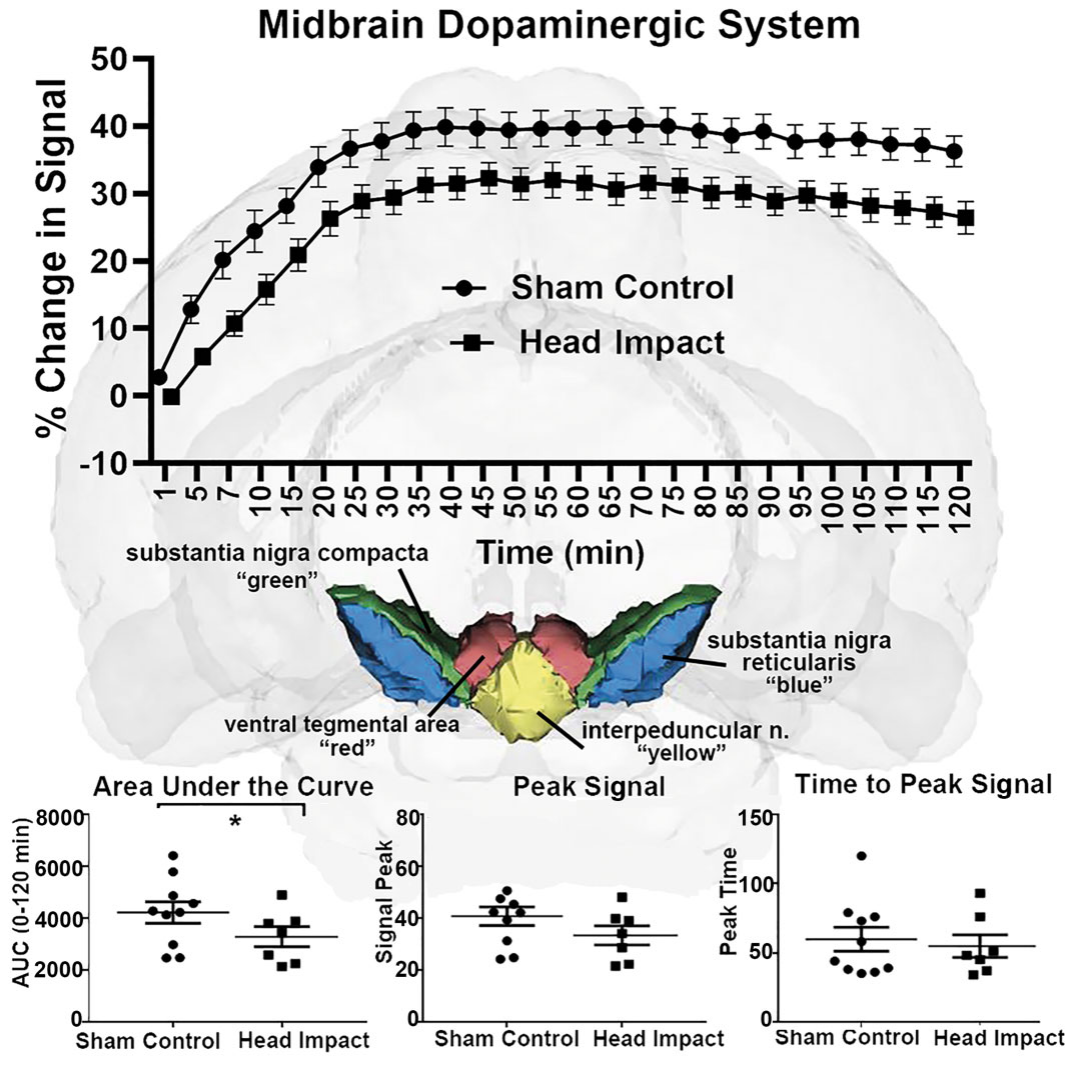
**Time-to-signal curve in midbrain dopaminergic system.** *Top panel*: Awake DCE-MRI shows the contrast agent kinetics following ICV injection over a 2-h period with data concatenated within the midbrain dopaminergic area. Two-way ANOVA identified a significant difference between the two groups [*P* = 0.0082, *F*(1,66) = 7.793]. Group sizes: Sham Control (*n* = 10); Head Impact (*n* = 7). *Middle panel*: The anatomical location of the midbrain dopaminergic system in the rat atlas, which is comprised of the substantia nigra compacta (green), substantia nigra reticularis (blue), interpeduncular nuclei (yellow) and ventral tegmental area (red). *Bottom panel*: Dot plots of extracted CA kinetic parameters, including AUC, peak signal, and time to peak signal for the sham control and head impact groups. The parameters extracted from the fitting curves were compared using two-sample *t*-test. *P < 0.05.

However, when looking at the composite of brain areas comprising the midbrain DA system (e.g. VTA, IPA, SN compacta and SN reticularis) there is a significant difference between sham control and head impacted rats for change in signal over time [*F*(1,66) = 6.551, *P* = 0.012] (Fig. 2). The AUC was significantly different (*P* = 0.019), there was a trend towards significance in peak signal (*P* = 0.055) but not time to peak (*P* = 0.548) (Fig. 2).

**Figure 3:**
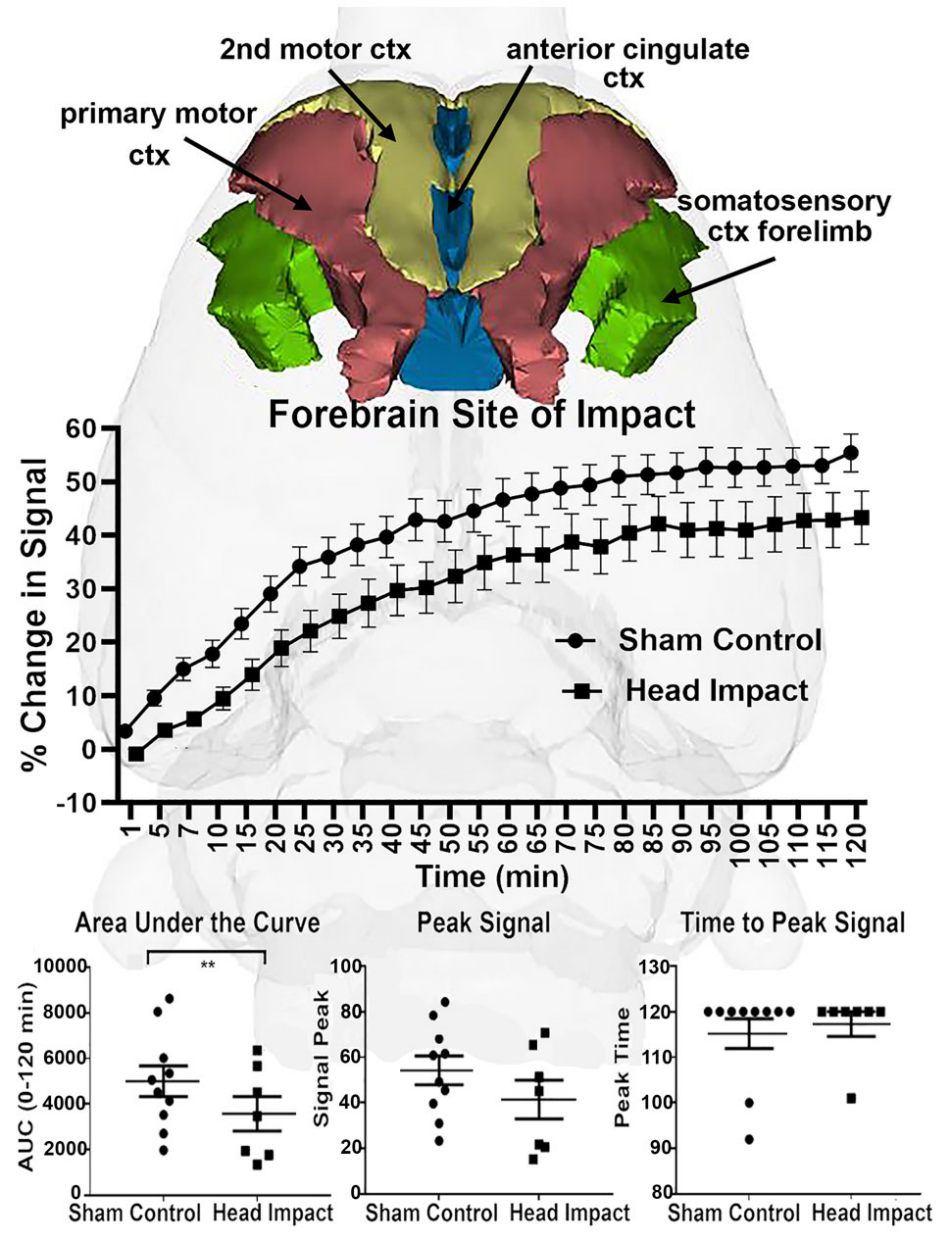
**Time-to-signal curve in the forebrain.** *Top panel*: The anatomical location of the forebrain in the rat atlas, which is comprised of the primary motor cortex (red), second motor cortex (yellow), anterior cingulate cortex (blue) and somatosensory cortex forelimb (green). *Middle panel*: Awake DCE-MRI shows the contrast agent kinetics following ICV injection over a 2-h period with data concatenated within the forebrain. Two-way ANOVA identified a tendency towards a significant difference between the two groups [*P* = 0.0673, *F*(1,66) = 3.462]. Group sizes: Sham Control (*n* = 10); Head Impact (*n* = 7). *Bottom panel*: Dot plots of extracted CA kinetic parameters, including AUC, peak signal and time to peak signal for the sham control and head impact groups. The parameters extracted from the fitting curves were compared using two-sample *t*-test. The head impact group showed significantly reduced CA transport (*P* = 0.0048, *t* = 2.921, df = 64). **P < 0.005.

The composite change in signal over time for the brain areas (e.g. anterior cingulate 2nd and primary motor cortices and somatosensory cortex hindlimb) in the impact area (see Supplementary Fig. 1A) are shown for both experimental conditions in Fig. 3. There was no significant difference between sham controls and head impacted rats for change in signal over time [*F*(1,66) = 3.462, *P* = 0.067]. The area under the curve (AUC) was significantly different (*P* = 0.048), there was a trend towards significance in peak signal (*P* = 0.052) but not time to peak (*P* = 0.829).

**Figure 4:**
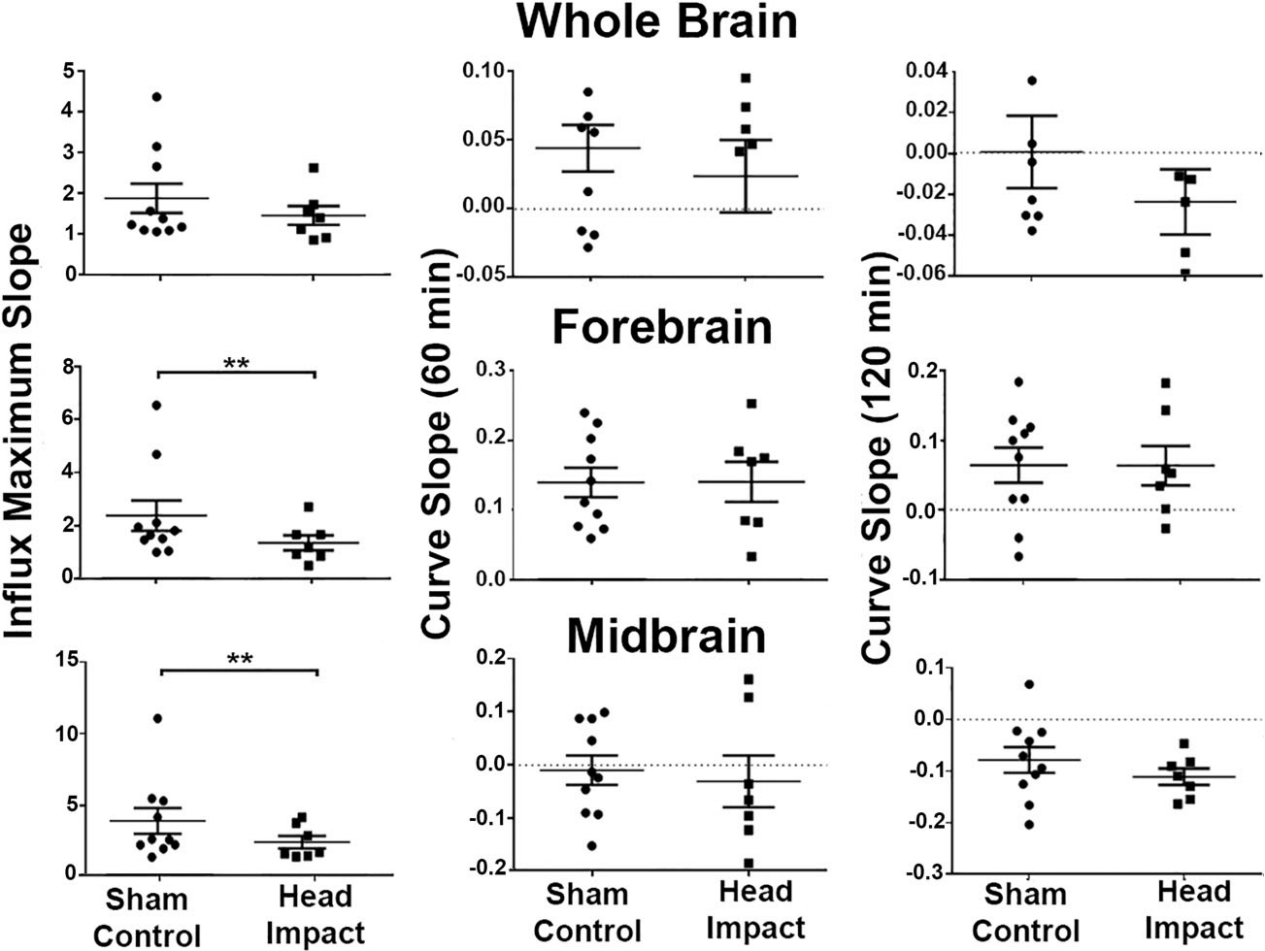
**Kinetics of contrast agent accumulation and flux.** Shown are dot plots for the whole brain, forebrain and midbrain highlighting the kinetics of the CA over the 120 min imaging period. The parameters extracted from the fitting curves were compared using two-sample *t*-test. **P < 0.01. Group sizes: Sham Control (*n* = 10); Head Impact (*n* = 7).

The kinetics of accumulation of CA in the whole brain, forebrain, and midbrain regions with and without head impact are shown in Fig. 4. For the whole brain, there were no significant differences in the slope of the kinetic at any phase of CA flux. The forebrain region at the site of head impact showed a significant difference in the influx of CA (*P* = 0.002). Rats with repetitive mild head impacts showed reduced influx with no difference in flux at 60 and 120 min as CA continued to accumulate in the area for both experimental conditions. The MDS showed a similar significant difference in influx of CA with head impacted rats presenting with a reduced influx as compared to sham controls (*P* = 0.008). However, unlike the forebrain there was a modest reduction CA levels at 60 and 120 min.

**Figure 5:**
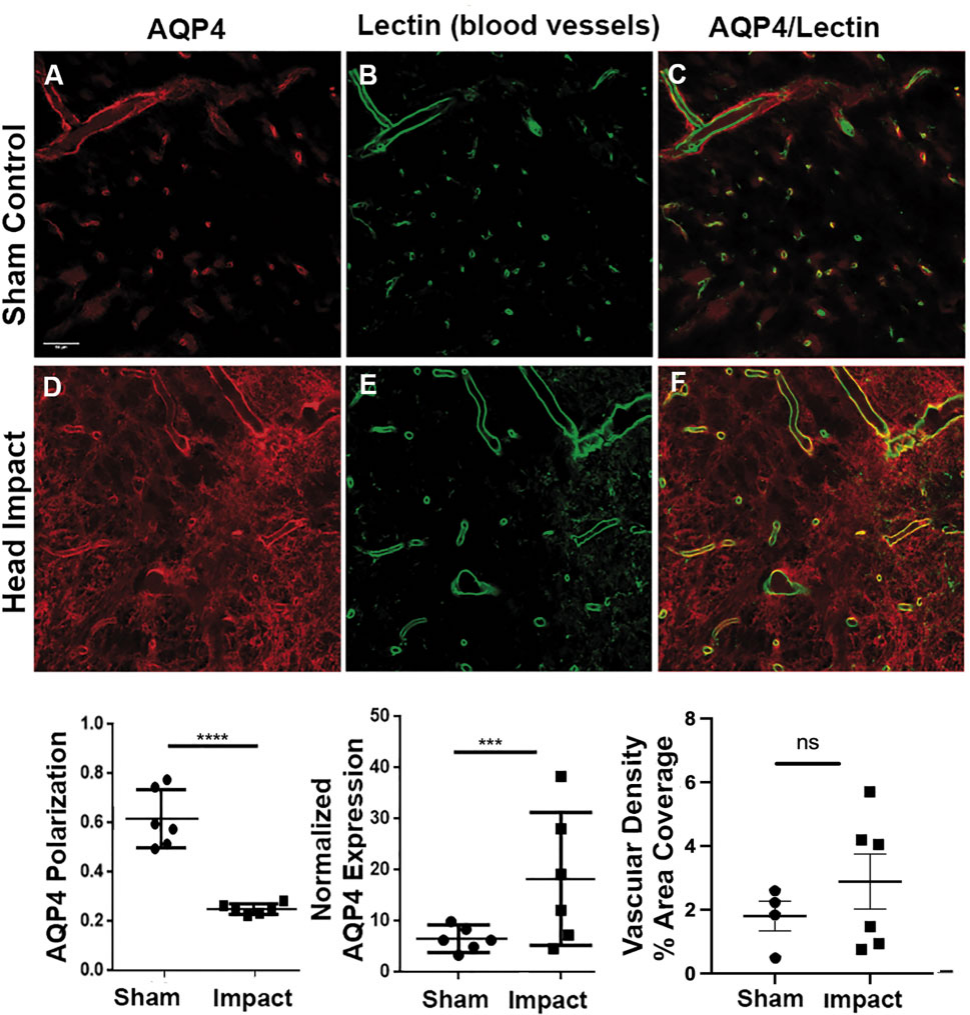
**Double immunofluorescence of aquaporin-4 (AQP4) protein (red) and blood vessels by lectin (green) in substantia nigra.** AQP4 polarity and AQP4 expression were quantified and compared by two-sample *t*-tests with *P* = 0.05 as the significance level (*n* = 6/group). Loss of AQP4 polarity (*P* < 0.0001) and a tendency towards increased AQP4 expression (*P* = 0.0811) is observed in the SN in head impact animals compared to controls. See the location of histology (left side) in Fig. 6. Values were compared using two-sample *t*-test. ***P < 0.001; ****P < 0.0001. Values are presented as dot plots. Scale bar: 50 µm.

Shown in Fig. 5 are double immunofluorescent images of AQP4 protein (red) and lectin-labeled blood vessels (green) in SN from sham controls (*n* = 6) and head impact rats (*n* = 6). These data came from brains harvested 3 weeks post impact to match the time course of the perivascular flux of trace summarized in Figs 1–3. AQP4 polarity was quantified as averaged perivascular AQP4/total AQP4. AQP4 expression was measured by perivascular AQP4 normalized to background intensity. Loss of AQP4 polarity (*P* < 0.0001) and an increased AQP4 expression (*P* = 0.0017) is observed in SN in head impact rats compared to sham controls. There was no significant change in vascular density.

**Figure 6:**
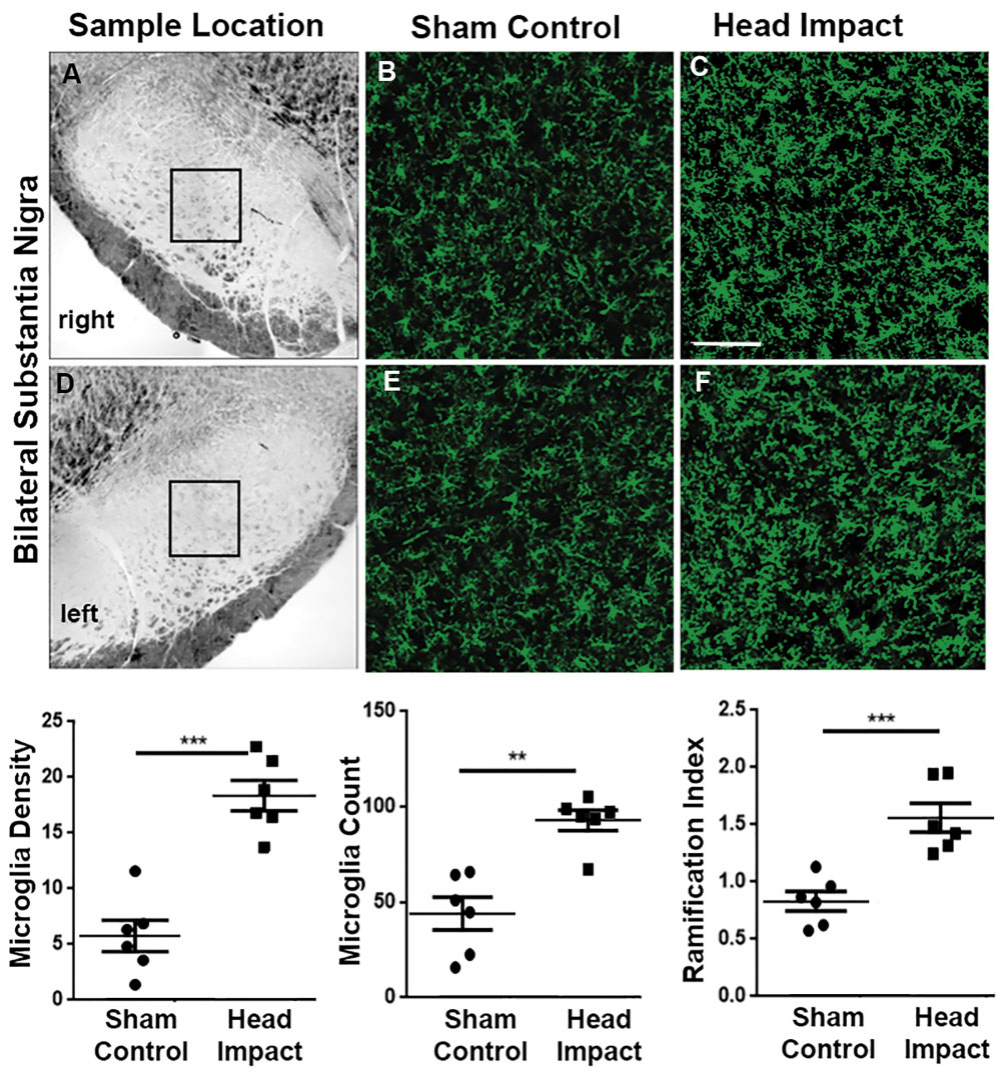
**Microglia immunohistochemistry in substantia nigra.** Iba1 staining was performed (*n* = 6/group) to investigate neuroinflammation. (**A**) Microglia in the bilateral substantia nigra (SN). Magnification: ×20. (**B**) Microglia density, measured by the fractional area of binary images within the ROIs, cell count per FOV, and ramification index carried out by Sholl analysis shows significant differences between the two groups (*P* = 0.0001, *P* = 0.0011, *P* = 0.0007) from two-sample *t*-tests. *P* < 0.05 is regarded as statistical significance. All values are shown as dot plots. **P < 0.01; ***P < 0.001; ****P < 0.0001. Scale bar: 100 µm.

**Figure 7:**
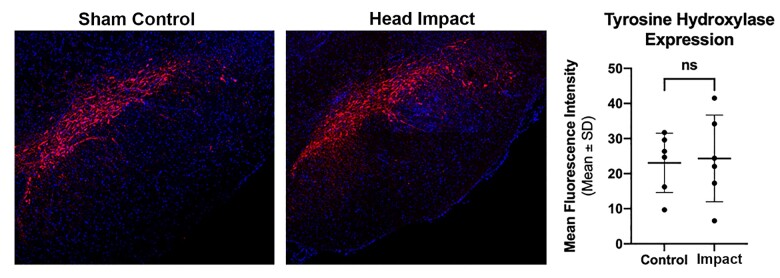
**Tyrosine hydroxylase staining.** Show in the panels is fluorescent staining (red) for tyrosine hydroxylase in the substantia nigra compacta. The blue fluorescence is DAPI (40,6-diamidino-2-phenylindole) staining used to identify cell nuclei. The mean fluorescent intensity (*n* = 6/group) for each experimental condition is shown in the dot plots. Values were compared using two-sample *t*-test. Scale bar: 100 µm.

Staining for activated microglia was performed to assess the level of neuroinflammation in the MDS following head impacts. Shown in the top right panels in Fig. 6 are the approximate location and dimension of the area (sample box) analysed from both sides of the SN. The green, fluorescent images show representative examples of microglia staining in a sham control and head impacted rat. Note the enlarged stellate structure characteristic of activated microglia. Dot plots summarizing the microglia density, measured by the fractional area of binary images within the sample box, cell count, and ramification index carried out by Sholl analysis, show significant difference between the sham controls and head impacted rats (*P* = 0.011, *P* = 0.001, *P* = 0.0007, respectively). There was no change in the level of TH staining in the SN between experimental groups (Fig. 7).

**Figure 8:**
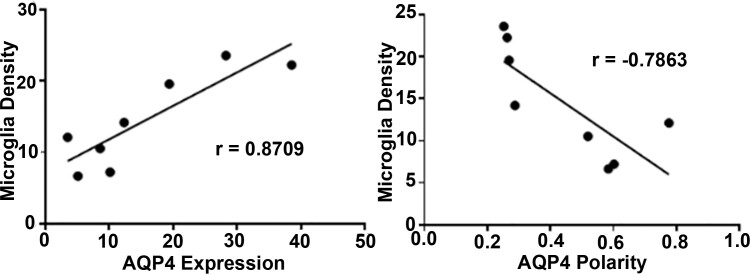
**Correlation analysis between AQP4 and microglia density in substantia nigra.** Microglia density is positively correlated to AQP4 protein expression (*r* = 0.8709) and negatively correlated to AQP4 polarity (*r* = −0.7863). *P* < 0.01 (*n* = 6/group).

There was a strong positive correlation between the density of microglia in the SN following head impact and the expression of AQP4 (*r* = 0.8709) (Fig. 8). There was also a strong negative correlation with microglia density and the polarity of AQP4 water channels (Fig. 8). This would suggest that neuroinflammation in the SN promotes the depolarization of AQP4 water channels away from the perivascular endfeet.

**Figure 9:**
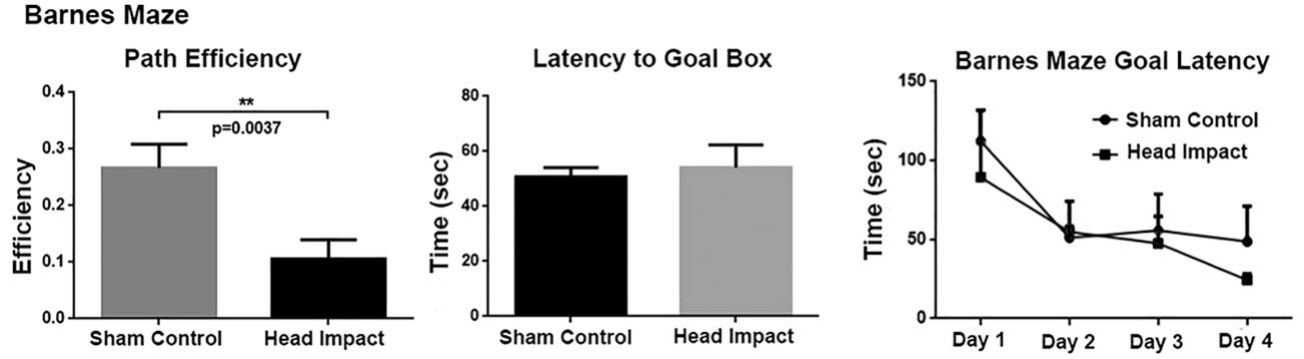
**Cognitive behaviour in the Barnes maze.** No significance is observed in escape latency to the goal box (*P* = 0.7034, *t* = 0.3809, df = 18), but head impact group showed a significantly decreased path efficiency (distance between the first and last locations divided by the total distance, *P* = 0.0037, *t* = 3.338, df = 18), **P < 0.005. There was no overall significant difference in two-way repeated-measures ANOVA on 4-day goal latency [*P* = 0.4781, *F*(1,18) = 0.5247, *n* = 10 sham control, *n* = 10 head impact].

Testing rats 5 weeks post head impact showed no overt differences in cognitive behaviour as measured in the Barnes Maze (Fig. 9). Escape latency to the goal box and path efficiency (distance between the first and last locations divided by the total distance) was averaged across trials and days to compare rmTBI rats to controls using two-sample *t*-tests. Path efficiency is significantly lower in rmTBI group (*P* = 0.0037). Latency to goal box was analysed to study the difference in spatial memory in the two groups of rats using two-way repeated-measures ANOVA with Sidak’s *post hoc* test for multiple comparisons.

## Discussion

This study was undertaken to test the hypothesis that mild repetitive head impacts would affect perivascular flow in the MDS. Indeed, two mild head impacts with no evidence of structural brain damage or contusion (Supplementary Fig. 1) caused a significant reduction in accumulation of ICV CA as compared to sham controls. In addition, the MDS showed extensive neuroinflammation as measured by an increase in putative microglia activation. These findings are discussed below with the respect to the measured increase in astrocytic AQP4 expression and depolarization that maybe promoting the efflux of water and toxic metabolites from the MDS as a neuroadaptive response to sustained vasogenic oedema.

In a recently published study, Cai et al.^19^ followed the kinetics of ICV CA across 171 brain areas in awake rats, reporting the peak signal was achieved within 30–35 min of infusion. In that study the SN showed a pronounced circadian variation in the flux of trace with the lowest levels occurring during the light phase of a 12:12 light–dark cycle at a time when rats are at rest or sleeping. This was interpreted as an increased efflux consistent with the notion that rest and sleep favour perivascular clearance of unwanted metabolites and proteins.^38^ The present studies looking at distribution of ICV CA in awake rats was also conducted during the light phase of the light-dark cycle with the added variable of repetitive mild head impacts. Twenty days post insult the collective midbrain dopaminergic areas of ventral tegmental area, interpeduncular n. and SN compacta and reticularis showed a similar profile of accumulation with CA peaking between 30–35 min but a significant reduction in the influx levels of trace in rats with a history of head impacts. Indeed, the slope of the CA influx and the level of CA accumulation were significantly reduced in head impacted rats as compared to sham control.

We propose two possible explanations for the reduced influx and accumulation of ICV CA in the MDS following head impact: (i) the neuroinflammation reflects an injury that may reduce perivascular flow to the area limiting access to the CA or (ii) the sustained neuroinflammation reflects a local environment of vasogenic oedema with enhanced clearance. The former cannot be excluded given there is less CA in head impacted rats both at the MDS and forebrain, the site of impact. CA infused into the lateral cerebroventricular would have to exit the ventricular system entering the subarachnoid space and down the perivascular space of penetrating pial vessels.^39^ This hypothesized circulation from the ventricular system to the perivascular microcirculation^40–42^ could be impeded in areas of neuroinflammation. Such a scenario would probably exacerbate the local problem, as decreasing perivascular flow would decrease the clearance of unwanted toxins and proteins at the site of injury. This response of the neurovascular unit to localized neuroinflammation would seem unreasonable particularly when considering the cause of the problem was attributed to two mild head impacts. The latter explanation of enhanced clearance is more plausible as an adaptive response to sustained localized neuroinflammation, a condition that promotes AQP4 expression and depolarization.^43^ As an adaptive response, the enhanced expression of AQP4 and movement of water channels along the astrocytic plasmalemma could favour efflux of water and solute back to the perivascular space aiding in increased clearance of CA. Indeed, the flux of water across AQP4 channels in astrocytic endfeet is bidirectional.^44^ The idea of enhanced clearance is consistent with the work from the Verkman lab showing vasogenic oedema from freeze injury was exacerbated in AQP4−/− mice as compared to wild type control.^45^ AQP4 water channels are needed to reduce the oedema from a leaky blood brain barrier at the site of injury. Freeze injury is reported to promote the formation of a AQP4 heteromultimeric water/ion channel complex that allow high capacitance transmembrane water transport in response to the localized oedema.^46^ This bulk water influx would be expected to raise the intracellular hydrostatic pressure swelling astrocytes and promoting efflux of water towards the capillary perivascular space. Despite the fact we did not stain for astrogliosis, this is a plausible explanation given the many studies on neuroinflammation and aquaporin depolarization along the astrocyte plasmalemma.

In a just published study, Christensen et al.^47^ reported on the perivascular clearance of CA in female adolescent rats subjected to three mild head impacts spaced three days apart. A 3 h long imaging session was conducted under anaesthesia, 2.5 h after injection of CA in the cisterna magna. The long duration between injection and data acquisition was necessary to establish a maximum and minimum signal intensity. A greater or lesser difference between signal would presuppose a change in the rate of clearance. Indeed, there was a purported decrease in clearance in limbic structures e.g. amygdala, hypothalamus, olfactory bulbs following mild repetitive head injury. Li et al.,^48^ using a comparable methodology to that described above, studied CA clearance 10 weeks after a single mild head injury in rats and reported a general decrease in clearance across much of the brain. It is difficult to reconcile these data reporting decreased clearance following mild head injury with our hypothesized increased clearance at the level of MDS. The methodologies are very different. We inject CA intraventricularly during the imaging session while rats are fully conscious and follow the kinetics of CA distribution that peak across the entire brain within 30–35 min. Whereas, these studies inject CA intracisternally via the cisterna magna and acquire data under anaesthesia delaying the kinetics of CA distribution by hrs.

In these studies, two mild head impacts, without evidence of structural brain damage or contusion, significantly increased the perivascular AQP4 expression and depolarization in the SN as measured 3 weeks following the last impact. Ren et al. using a mild, closed head impact model ‘Hit and Run’ in mice, reported depolarization of AQP4 in the cortex and striatum. In these studies, the mild, single head impact presented with no signs of structural brain damage and the changes in AQP4 depolarization resolved with 14–28 days. The impact was directed towards the side of the head and analysis of perivascular AQP4 was confined to the cortex and striatum. Reactive astrogliosis and microgliosis was observed at the site of cortical impact, striatum, and hippocampus at three days post impact. Cortical gliosis did not resolve until 14–28 days post impact. The authors noted that the transient increase in neuroinflammation and relocalization of AQP4 water channels along the astrocytic processes away from the perivascular space could be a compensatory mechanism to reduce cerebral oedema and changes in intracerebral pressure. Both the Ren study and ours focus on mild head impact without structural brain injury and underscore the loss of perivascular AQP4 polarization together with enhanced neuroinflammation at site-specific brain areas. Ren et al., viewed this as an adaptive response of astrocytes to chronic neuroinflammation and sustained vasogenic oedema that may occur with injury to the blood brain barrier. In a just published study, we showed a single mild impact using the same close-head momentum exchange model causes a transient heterogeneous increase in vasogenic oedema as measured by an increase in apparent diffusion coefficient. The increase in extracellular fluid volume peaked at 6 h but returned to baseline by 24 h. In this study using two head impacts separated by 48 h, we report a sustained neuroinflammation at the level of MDS without any significant changes in TH staining 3 weeks post insult.

When tested for learning and memory using the Barnes maze, head impacted rats showed no gross cognitive deficits. The decrease in path efficiency in the Barnes maze of impacted rats was the only measure of note. The absence of any gross changes in cognitive behaviour at 3 weeks following mild head impact is not surprising given the many studies that report resolution of motor and cognitive dysfunction within days of mild head injury.^49–51^ In a previous study from our lab following the neuroradiology and behaviour effects of three mild head impacts separated by 48 h there were no signs of motor or cognitive dysfunction 9 weeks post insult.^15^

Moderate to severe TBI is a risk factor for Alzheimer’s,^52^^,^^53^ Parkinson’s^4^ and Amyotrophic Lateral Sclerosis.^54^ However, mild head injury as modelled in this study, is the most prevalent form of head injury and is estimated to affect over 42 million people worldwide every year due mostly to falls and motor accidents.^55^^,^^56^ The health community lists guidelines for diagnosing these mild head injuries that include self-reports of transient confusion, disorientation, impaired consciousness, or dysfunction in memory around the time of the injury and, importantly, no apparent structural damage as determined with imaging.^56^^,^^57^ Repetitive mild head impacts in organized sports or on the battlefield have raised concerns about their long-term effects on neurodegeneration, e.g. CTE.^58^ Veterans with a history of mild head injury have a 56% higher risk of developing Parkinson’s disease with ageing.^7^ There are numerous human and animal studies using various in-vivo and in-vitro methods showing that repetitive mild head injury separated by short intervals of time posse a significant risk to the brain and vulnerability to neurodegenerative disorders with ageing.^59–62^

Early epidemiological studies weighing the risk of concussions with the risk of developing Parkinson’s disease were equivocal. Several studies report no clear evidence showing causality between head injury and later Parkinson’s disease.^63–65^ The issue of reverse causation, i.e. early prodromal Parkinson’s disease may increase risk of head injury, was addressed in a study of Taylor and colleagues who reported an association for risk of Parkinson’s disease with head injury at least 10 years prior to diagnosis of Parkinson’s disease.^6^ Another study using meta-analysis also reported an association with a history of head trauma and Parkinson’s disease.^4^ In a case control study of 196 subjects who developed Parkinson’s disease, the frequency of head injury was significantly higher in Parkinson’s disease patients than controls matched for age and gender.^66^ The most compelling epidemiological study to date linking head injury to Parkinson’s disease is a prospective study on over 7000 subjects with neuropathology data following autopsy. In this study, head injury with loss of consciousness was associated with risk for Lewy body accumulation, progression of parkinsonism, and Parkinson’s disease, but not with dementia, Alzheimer’s, neuritic plaques, or neurofibrillary tangles.^3^

### Limitations

This was study was limited to male rats and a single time point for analysis, 3 weeks post insult. There is an ever-expanding literature reporting sex differences in response to mild head injury.^67–71^ Caplan et al., in a recent review of the literature, provides a compelling argument supporting male/female differences in morbidity and mortality following head injury.^72^ There is also a sex differences in Parkinson’s disease as it is more prevalent in men than women.^73^ While Parkinson’s disease is more prevalent in males and the incident of head injury occurs more often in young males, because of sports and risk taking activates, there are no preclinical or clinical data to suggest perivascular flow will be any different between males and females following head injury. The overarching goal of this research was to understand risk factors contributing to Parkinson’s disease. We hypothesized that mild repetitive head impact would be a contributing factor to the aetiology and pathophysiology of Parkinson’s disease. There was no change in TH levels in the SN at 3 weeks post insult and we did not stain for α-synuclein or Lewy bodies. While the evidence of microgliosis and AQP4 expression portends of future problems, it would have been necessary to follow rats to end-of-life (ca. 2.5 years) looking at the major contribution of ageing in Parkinson’s disease.

### Summary

Data show that just two mild head impacts separated by 48 h, increase microgliosis and AQP4 depolarization and expression along capillaries in the midbrain dopaminergic system. This area of inflammation has a reduced accumulation of imaging contrast agent suggesting an increase in perivascular clearance to compensate for localized oedema. These findings support the concerns raised by the healthcare community regarding the long-term consequences of repetitive mild head injuries as an early risk factor for senile Parkinson’s disease for participants in organized contact sports and military personnel.

## Supplementary material

Supplementary material is available at *Brain Communications* online.

## Funding

This work was supported by an American Heart Association pre-doctoral fellowship (18PRE33960461), National Institutes of Health grant (K01-HL125499).

## Competing interests

C.F.F. has a financial interest in Animal Imaging Research, the company that makes the RF electronics and holders for animal imaging.

## Supplementary Material

fcab265_Supplementary_Data

## Abbreviations

AQP4: aquaporin 4
AUC: area under the curve
CA: contrast agent
CSF-ISF: cerebrospinal fluid and interstitial fluid
DA: dopamine
EPI: echo planar imaging
ICV: intracerebroventricular
IPN: interpeduncular nucleus
Iba1: ionized calcium binding adaptor molecule 1
MDS: midbrain dopaminergic system
NOP: novel object preference
PFC: prefrontal cortex
RARE: rapid acquisition relaxation enhanced
SN: substantia nigra
TBI: traumatic brain injury
TE: echo time
TH: tyrosine hydroxylase
TR: repetition time
VTA: ventral tegmental area
