# Supplementary material for: Mild repetitive head impacts alter perivascular flow in the midbrain dopaminergic system in awake rats
Source: Brain Commun. 2021 Nov 3;3(4):fcab265. doi: 10.1093/braincomms/fcab265 (PMC8600963; doi:10.1093/braincomms/fcab265)

**Supplementary Figure 1.** Absence of structural brain damage or contusion with two mild head impacts

Shown in panel A is a representative example from a single rat showing the site of impact extending across three contiguous sections and the edema (arrow) over the soft tissue above the skull observed within hrs of a mild head impact. Panel B are two representative examples, rats R2 and R3, of axial neuroanatomical images extending rostral (top) to caudal (bottom) taken three weeks following two mild head impacts. Panel C shows example of sham control (no hit) at the level of the forebrain.


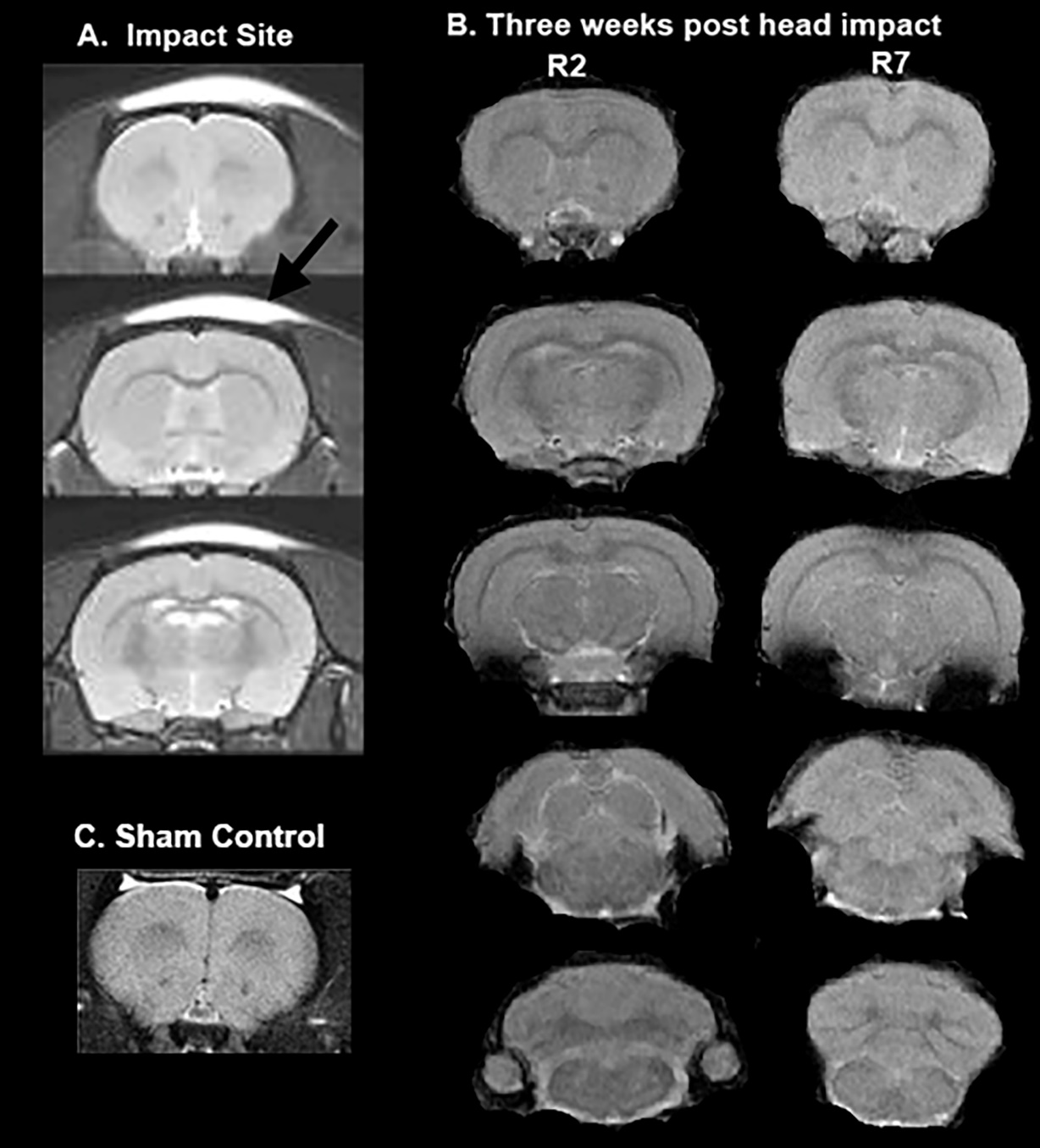

Supplement: fcab265_Supplementary_Data [file fcab265_supplementary_data.docx]
